# Development of a Novel Water Jet Polisher Using Soft Abrasives for Small Complex-Structure Heat Pipes of Aluminum Alloy Produced Using Additive Manufacturing

**DOI:** 10.3390/ma17030582

**Published:** 2024-01-25

**Authors:** Tianyu Zhang, Zhenyu Zhang, Junyuan Feng, Chunjing Shi, Hongxiu Zhou, Fanning Meng, Dingyi Tong

**Affiliations:** 1 State Key Laboratory of High-Performance Precision Manufacturing, Dalian University of Technology, Dalian 116024, China; 2 School of Mechanical Engineering, Hangzhou Dianzi University, Hangzhou 310018, China; 3 School of Mechanical and Electrical Engineering, Hainan University, Haikou 570228, China; 4 School of Energy and Power Engineering, Dalian University of Technology, Dalian 116024, China

**Keywords:** water jet polishing, additive manufacturing, complex structure, small size, Al alloy

## Abstract

It is a challenge to polish the interior surface of a small bent pipe with complex structures and sizes less than 0.5 mm. This is because of the fact that traditional polishing methods could destroy, block, or break the small complex structures. For a small bent pipe made of aluminum alloy produced using additive manufacturing, the defects, such as adhered powders and spatters, are easy to jam the pipe without polishing, possibly resulting in catastrophic failure for aerospace applications. To overcome this challenge, a novel water jet polisher was developed using soft polymethyl methacrylate (PMMA) abrasives. After polishing a specific area, the adhered powders on the interior surface were reduced from over 140 to 2, 3, and 6 by the soft abrasives with mesh sizes of 200, 400, and 600, respectively. The surface roughness Sa was decreased from 3.41 to 0.92 μm after polishing using PMMA abrasives with a mesh size of 200. In comparison, silica abrasives were also employed to polish the small bent pipes, leading to the bent part of pipes breaking. However, this kind of failure was absent when using soft abrasives. Computational fluid dynamics calculations elucidate that a peak erosion rate of silica abrasives for a bent pipe with a turn angle of 30° is 2.18 kg/(m^2^·s), which is 17 times that of soft abrasives. This is why the small bent pipe was broken using silica abrasives, whereas it remained intact when polished with soft abrasives. In addition, water jet polishing has a lower erosion rate, a relatively smooth erosion curve, and less erosion energy, leaving the bent parts intact. The developed soft abrasive water jet polisher and the findings of this study suggest new possibilities for cleaning the adhered powders and spatters and polishing the interior surface of small bent pipes with complex structures.

## 1. Introduction

A heat pipe pipe is an essential component in aerospace devices for effective heat transfer. However, the design of the actual cooling system and the geometric structure of the served device often result in a complex spatial shape for heat pipes. Additive manufacturing technology provides new possibilities for manufacturing many complex structures [1,2,3,4,5,6]. The production of heat pipes using additivie manufacturing has garnered considerable attention due to its advantages in processing complex and highly customizable parts, integrated manufacturing with structural components, lightweight design, and high processing efficiency [7,8,9,10,11]. Unfortunately, the surfaces of parts manufactured using additive manufacturing are accompanied by a large amount of scattered or slightly adhered powder, which significantly affects the surface quality of the printed parts and the practical stability of the heat pipe flow channel [12,13,14]. During the launch and maneuver of the space equipment, the influence of acceleration and thermal stress can lead to the falling off and aggregation of loose powders, which could potentially lead to channel blockage, resulting in a decline in the performance of the equipment and even the total failure of the overall device. In addition, excess materials need to be removed to save on the overall launch price. Therefore, it is necessary to investigate how to efficiently remove satellite structures in the additively manufactured heat pipes.

In recent years, jet polishing, as an efficient and environmentally friendly surface treatment technique, has been widely adopted in the field of treating various complex workpieces [15,16,17,18,19,20]. Zhao et al. [21] combined Taguchi’s method with response surface technology to design a rotating jet polishing method, and carried out jet polishing experiments on the inner wall of 304 stainless steel round tubes with a diameter of 50 mm. After polishing, the average surface roughness of the inner wall was reduced from 1.05 μm to 0.212 μm. Feng et al. [22] conducted an experimental study on a straight heat pipe with a length of 400 mm using a multiphase jet polishing method, which demonstrated the effectiveness of directly treating the inner wall of the pipe from the inlet jet.

However, it is obvious that when the fluid carrying solid particles flows through the bend, it will inevitably cause erosion on the wall, especially in the area where the flow field distribution is concentrated, and the effects of this erosion are also magnified by the wear particles traditionally used [23,24,25,26,27,28]. Therefore, there is an urgent need to develop new non-erosive or weak erosion abrasives in this application field. In this regard, the study of novel abrasive particles has received a lot of attention. Ke et al. [29] used a new type of composite abrasive with different diameters of SiC particles coated with PS polymer for the jet polishing of silicon wafers, shortening the post-processing time required for silicon wafer regeneration. Yang et al. [30] used dry ice particles to carry out jet experiments with different parameters on the surface of stainless steel electrode, and the roughness of the samples decreased from 0.0708 μm to 0.0395 μm after polishing. Zhu et al. [31] used medium-hardness amino thermosetting plastic particles for abrasive gas jet polishing. A series of polishing experiments were carried out on the surface of the rolled slab of a massive 7075 aluminum alloy, which verified the feasibility of reducing hardness to reduce abrasive embedding.

In theory, some of these abrasive particles can reduce the erosion effect. However, the researchers in these papers only conducted verification experiments on the surfaces of common workpieces. The above-mentioned research still primarily focused on the surface of the part or slightly larger sized studies. The researchers did not systematically analyze and study the processing parameters and results, and they did not use weak erosion abrasive particles to treat the additive heat pipe with an angle. Building upon the preceding information, this study utilized a self-built abrasive water jet (AWJ) equipment to clean the AlSi10Mg alloy heat pipe manufactured through additive processes. The investigation focused on understanding the mechanism of corner erosion induced by a commercial SiO_2_ abrasive and two types of plastic abrasives. Then, the cleaning efficiency, the material removal rate, and the resultant surface roughness using PMMA and polystyrene (PS) abrasives were thoroughly investigated. Furthermore, considering that it is difficult to directly monitor the velocity and trajectory of abrasive particles in pipe treatment experiments, computational fluid dynamics (CFD) simulations were also conducted to study the mechanisms of the cleaning process. The energy loss, the impact frequency, and the erosion rate of both SiO_2_ and plastic abrasive water jet cleaning were compared and analyzed.

## 2. Experimental Preparation

### 2.1. Additive Manufacturing of Heat Pipes

**Figure 1:**
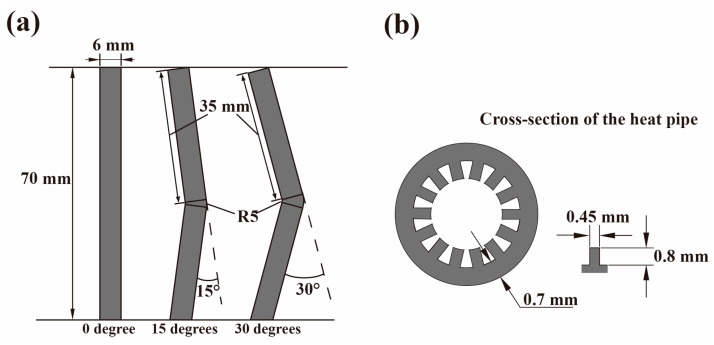
The shape and parameters for the heat pipe:(**a**) side elevation; (**b**) cross-section.

Figure 1 shows the cross-section topography and shape parameters of the heat pipe used in the experiment. For all heat pipes, the infill scanning speed was 900 mm/s and the laser power was 135 W; detailed print parameters are listed in Table 1. AlSi10Mg alloy powder ranging in size from 15 to 53 μm was bought from Jiangsu Vilory Advanced Materials Technology (Xuzhou, China) for this investigation.

### 2.2. Experimental Equipment and Operation

**Figure 2:**
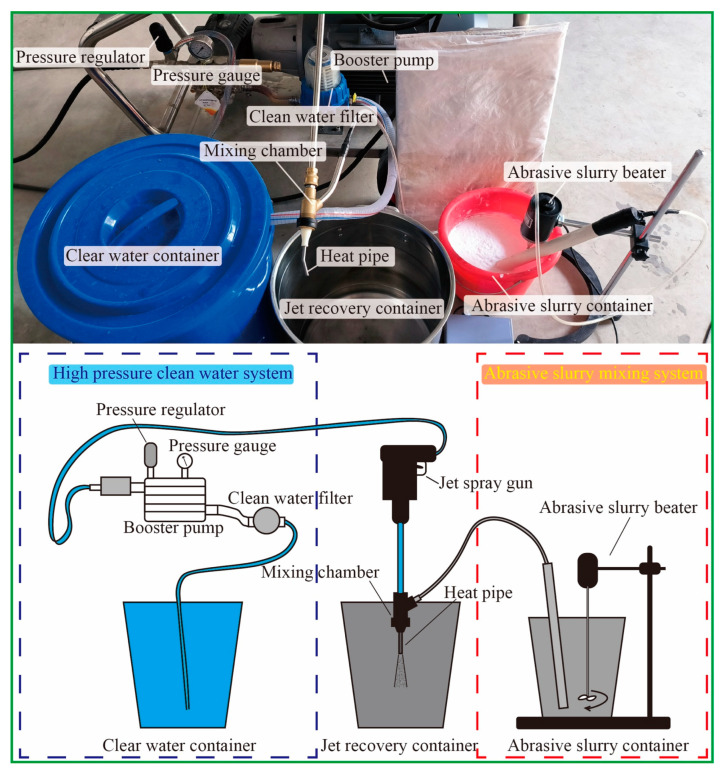
Equipment and schematics used in the experiment.

The experimental parameters were determined through pre-experimentation and the specifications of the equipment, ensuring the effective polishing of the heat pipe. The flow rate was determined based on the pump pressure after conducting actual tests to achieve optimal results. A 0–40 MPa power washing pump, as shown in Figure 2, was used as drive for the abrasive water jet. All three types of abrasive were first mixed with pure water inside the mixing tank, with a 15 wt.% concentration. Then, the slurry was sucked into the mixing chamber through a pipe, and the negative pressure was provided by the high-speed water pumped by the power washing pump. After the slurry and the pure water were mixed, they left the chamber together forming a water jet with the abrasive inside it. To connect the heat pipe to the nozzle, a 3D printed adapter was printed and used. During the cleaning process, the abrasive slurry exited the heat pipe and was collected inside a large tank; it was then recycled after a long settling time. Information on the jet pressure abrasive particles used in the experiment is listed in Table 2. A 30 MPa pressure was used for all experiments and the flow rate of both the pure water as well as the slurry was measured under 30 MPa. Since the ratio of the flow rate of the pure water and slurry was 1:10, the final concentration of abrasive in the jet was calculated as 1.5 wt.%. For the selection of abrasive particle size, considering the width of the heat pipe groove (0.45 mm) and height (0.8 mm), the narrowest space between the grooves in the circumferential direction of the heat pipe is about 0.22 mm, and the widest space is approximately 0.58 mm. To enable the abrasive particles to effectively remove the floating powder and unstable structure within the groove gap, the standard actual size of the maximum abrasive particle size #600 was about 23 μm, while that of #200 was about 70 μm. This setting maintains the particle size of the abrasive particles at about 1/10 of the gap, ensuring that the abrasive particles can easily reach every position of the heat pipe. Simultaneously, the size of the AlSi10Mg floating powder is 10–53 μm, and the particle size of the abrasive particles is similar in volume, making it difficult to form blockages while ensuring effective removal. To explore the influence of different particle sizes on removal results, #400 was also introduced as an intermediate value to create a particle size gradient for a more comprehensive experiment. The SiO_2_ abrasive used in the experiment was from Gongyi Xinde Water Purification Materials Sales Co., LTD (Zhengzhou, China), and the PMMA and PS abrasive were from CHIMEI Corporation (Tainan City, China); information on the abrasive particles is shown in Table 3. Each abrasive particle has three particle sizes: #200, #400, and #600.

Although the same batch of heat pipes printed with the same printing parameters was used in the experiment, the initial mass of each heat pipe was not the same. Therefore, each heat pipe measured the mass change separately. Before weighing, the heat pipe was rinsed with clean running water and ultrasonically cleaned for 3 min, then dried with compressed air. Because the SiO_2_ abrasive particles were penetrated too early in the preliminary experiment, we adopted different strategies for polishing experiments with different abrasive particles. For the three sizes of heat pipes treated with SiO_2_ particles, the straight pipes were cleaned using the abrasive water jet for 1 min, and the mass before and after that was recorded using a precision balance. The 15° and 30° bent pipes were treated with the abrasive water jet until they leaked, and the time as well as the weight when leaked were recorded. For the PMMA and PS abrasive-treated heat pipes, each heat pipe was cleaned using the abrasive water jet for a total of 10 min. During the experiment, the heat pipe was weighted every 2 min, and afterwards, they were ultrasonically cleaned and high-pressure air-dried. Detailed information on the full-factorial experiments can be found in Table A1 and Table A2. After the experiment, all heat pipes were sawed along the axial direction using a diamond wire cutting machine, which facilitated the observation and characterization of the treated surface by means of an optical microscope (BX53M, Olympus, Tokyo, Japan), an optical surface profiler (Zygo NewView^TM^ 9000, Middlefield, CT, USA), and SEM (JSM-7800F, Tokyo, Japan).

### 2.3. CFD Simulation

**Figure 3:**
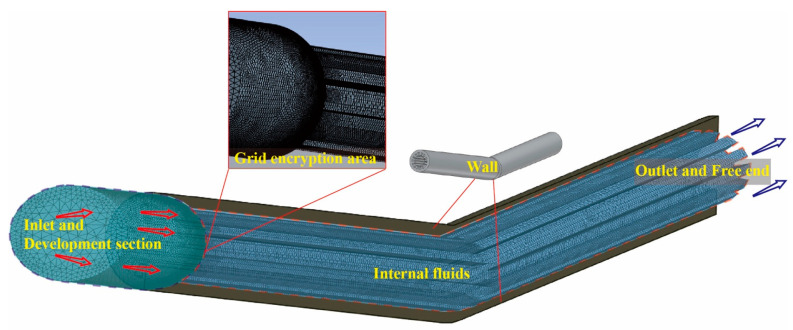
Model diagram for the simulation.

The 15° 3D model in the CFD simulation is illustrated in Figure 3. A 10 mm long cylindrical area was applied at the entrance as a transition zone for the development of the flow field, and the grid at the connection was refined. The modeling method for the 30° tube was in alignment with this. The Fluent fluid flow module of ANSYS was chosen for the CFD simulations, and abrasives were treated as discrete phases by the DPM module. The particle density, size, and shape were set, respectively, according to the material properties of the actual abrasives in this study. The pressure inlet was set to 30 MPa, with the discrete phase boundary type as ‘escape’. The pressure outlet was set to 1 MPa, also with the discrete phase boundary type as ‘escape’. The wall was defined as a non-slip stationary wall, with the discrete phase boundary type as ‘reflect’, ignoring the influence of heat. The surface roughness was set to 0.005 mm, based on the actual roughness of the additive manufacturing component. The wall reflection of particles was set as a function of incident angle, where the ratio v_2_/v_1_ ranges from 0.2 to 0.8 according to a previous study. Due to the angular characteristics of the inner wall jet impacting the wall surface, the Finnie wall erosion model was used to calculate the erosion rate [26,32,33,34,35,36]. Abrasives were injected into the region with a cone shape, where the spread angle was 30° and the initial velocity was 10 m/s. The material parameters were set according to the actual properties, and the problem was solved using the coupled solution scheme with default spatial discretization settings. After the simulation, the fluid velocity field and the particle status were both extracted and analyzed. The impact location, the impact speed, as well as the impact angle of all particles were all calculated using a MATLAB script we wrote.

## 3. Results and Discussion

### 3.1. Commercial SiO_2_ Abrasive Particles for the Erosion Effects and Analysis of High-Pressure Water Jets

**Figure 4:**
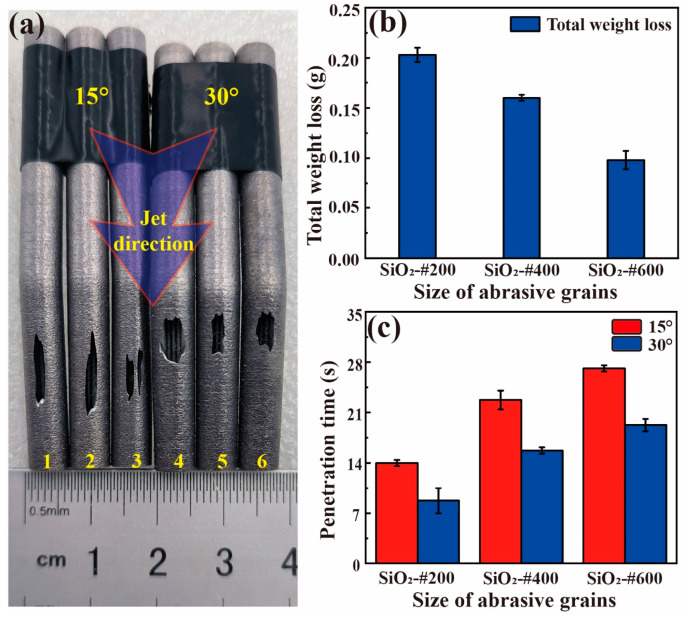
Erosion caused by the SiO_2_ abrasive particles: (**a**) erosion locations of the heat pipes, (**b**) total weight loss of the 0° heat pipe, and (**c**) penetration time of the 15° and 30° heat pipes.

As shown in Figure 4, all heat pipes treated with SiO_2_ abrasive particles were seriously eroded and leaking holes on the wall were found. Though the shape of the leaking holes was different, their location for different bent angles was similar. From Figure 4b, it can be seen that the total weight loss gradually decreases as the abrasive size becomes smaller, showing that the capability of material removal is positively proportional to the abrasive size. As a result, the penetration time before the leak also increases as the particle size decreases, and a larger bent angle also achieves the penetration of the wall sooner.

**Figure 5:**
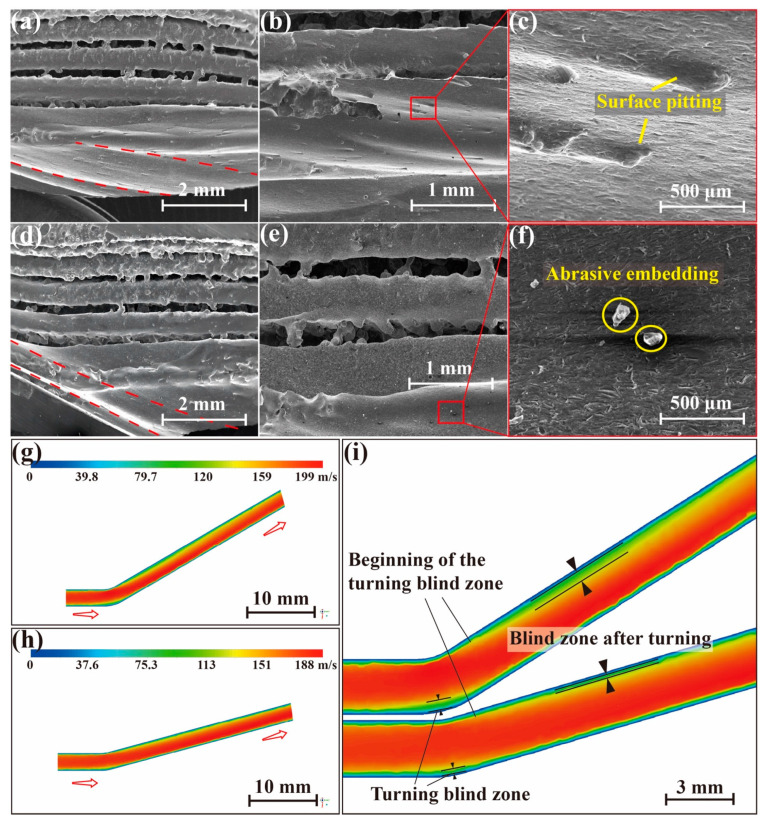
Corner of heat pipe after SiO_2_ abrasive treatment: the erosion morphology of the corner of the 15° heat pipe (**a**–**c**) and the 30° heat pipe (**d**–**f**); the flow field distribution obtained by simulation for the 30° heat pipe (**g**) and the 15° heat pipe (**h**); and jet blind zone position (**i**).

As can be seen from Figure 5, the erosion area of the 15° and 30° heat pipes is slightly different, and the microstructure and wall of the heat pipe are eroded and a channel is carved along the way. It is worth noting that the angle marked with the red line (Direction of heat pipe wall) exactly matches the bent angle of the heat pipe. The upper part and the lower part of the heat pipe, as shown in Figure 5a,d, have very different morphology. Many satellites and molten balls still exist above the cross-section of the heat pipe, while the bottom has been eroded to a smooth or sprayed finish. The embedding of the abrasive particles and the pitting of the surface are observed from Figure 5b,e. The result of the CFD simulation is displayed in Figure 5i, and the velocity field shows that a blind zone exists on the upper part of the heat pipe after the turning. And the blind zone is larger when the bent angle is larger, which matches the actual result.

Based on the above analysis, it is evident that conventional SiO_2_ abrasive particles induce significant erosion on the inner walls of the heat pipe. Due to its high removal rate, not only was the balling microstructure removed, but also the original micro groove as well as the wall were damaged. It should be noted that the penetration time is only a dozen seconds, so traditional hard abrasives are unsuitable for cleaning curved heat pipes. Given that the defects targeted for removal in this work are loosely connected to the surface of the satellite, the required material removal rate is much lower than that of the SiO_2_ abrasive.

### 3.2. Plastic Abrasive Particles Are Used for the Treatment Effect and Analysis of High-Pressure Water Jets

**Figure 6:**
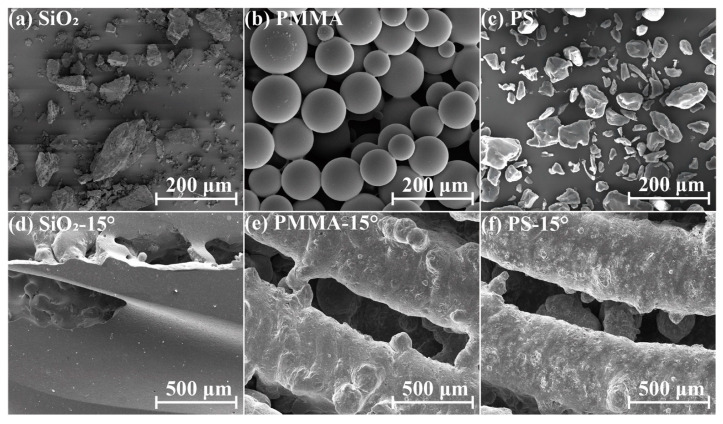
Morphology of the abrasive particles and preliminary experimental results: (**a**) the commercial SiO_2_ abrasives, (**b**) the PMMA abrasives, (**c**) the PS abrasives, the corner interior of the 15 degrees bent pipe polished by (**d**) commercial SiO_2_ abrasives, (**e**) PMMA abrasives and (**f**) PS abrasives.

Based on the previous unsuccessful cleaning experiments, the abrasive should be hard and dense enough to remove unstable satellites but should not cause erosion at the corners. According to the studies of Zhu et al. [31] and Kim et al. [34] on the influence of physical properties such as the hardness and sharpness of the abrasive particles on jet material removal, the hardness as well as the density of the abrasives are two important factors. Under the same conditions, they are positively correlated with the degree of erosion. In addition, the shape of the abrasive is also important because sharper edges can be more likely to damage the material. Based on the above, we decided to use high-hardness plastic abrasive particles instead of SiO_2_ abrasive particles to reduce or prevent the occurrence of this erosion phenomenon. Figure 6 shows the SEM image of the three types of abrasive particles. The shapes of both PS and SiO_2_ are irregular, while the PMMA is of spherical shape. As shown in Table 3, though the shape is similar, the density of PS is only half that of SiO_2_, and the hardness is only about 1/3. The density of the PMMA abrasive is similar to that of the PS abrasive, but the hardness is higher. Therefore, though the impingement intensity of both plastic abrasives should be similar, the material removal rate should be different. A preliminary experiment was conducted on the removal morphology of the three abrasives. As shown in Figure 6d–f, the heat pipe treated with the SiO_2_ abrasive particles was completely sprayed, while the heat pipe treated with the plastic abrasive particles did not show a serious erosion morphology.

**Figure 7:**
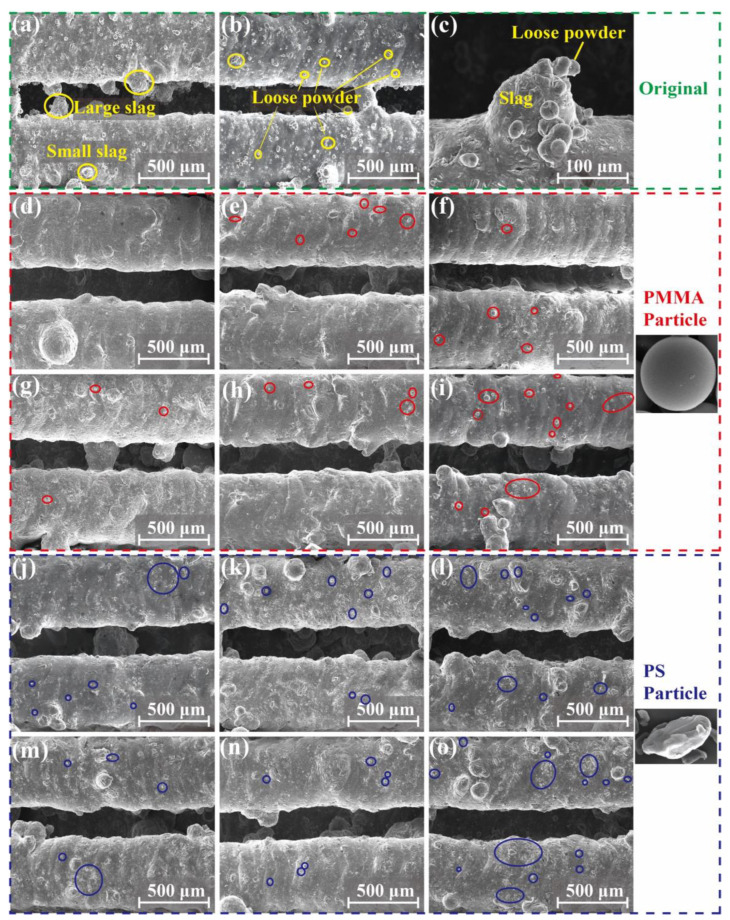
Morphology of the straight heat pipe after plastic abrasive treatment: (**a**–**c**) original surface; (**d**,**j**) #200, (**e**,**k**) #400, and (**f**,**l**) #600 are inlet; (**g**,**m**) #200, (**h**,**n**) #400, and (**i**,**o**) #600 are outlet.

**Figure 8:**
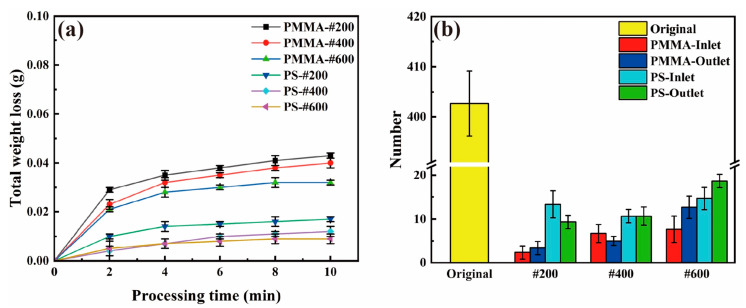
Straight heat pipe after plastic abrasive treatment: (**a**) curves of mass loss, (**b**) the amount of residual structure.

To quantitatively study the effectiveness of the AWJ cleaning method with plastic abrasives, we first conducted the AWJ cleaning experiment on straight heat pipes. Figure 7 shows the surface morphology of the inlet and outlet of the heat pipe before and after AWJ treatment. Before the AWJ cleaning, the surface was filled with small particles called satellites, which are unmelt or half-melted printing powders. Spattering powders are also another form of satellites. As shown in Figure 7a–c, these particles are at the scale of 0.03–0.05 mm, which are much smaller than the balling found on the surface. As shown in Figure 7d–o, after the cleaning with the PMMA and PS abrasives, most of the satellites were removed. Three regions of 1.25 × 1.5 mm were randomly selected at different positions on the inner surface of the treated heat pipe, and the number of remaining satellites were counted. The circled area represents the location of the remaining powders, and the statistical results are utilized as one of the evaluation indices for cleaning ability. As shown in Figure 7d, the inlet of the heat pipe cleaned using PMMA-#200 was free of satellites, but there were still a few satellites left on the #400 and #600 polished group. For the PS group, even the #200 group is still not completely cleaned, which matches the material removal volume result. The surface of the heat pipe treated with PMMA abrasives was significantly better, and the amount of residual microstructures on the surface was also smaller than that of the PS abrasive-treated heat pipe. In the comparison of the removal effects of the #400 and #600 abrasives, the effect of the PMMA abrasive treatment is also better than that of the PS abrasives, with the total removal of PS abrasives of sizes #400 to #600 being essentially around 0.01 g. According to the SEM morphology in Figure 7, the inner surface of the heat pipe treated with PS particles of sizes #400 to #600 is relatively poor, and there is still a large amount of floating powder that has not been cleaned. As shown in Figure 8b, the number of satellites processed using PMMA abrasives can be reduced from 400 to fewer than 10, representing only 1/40 of its original count. The number of satellites processed using PS abrasives can also be reduced to fewr than 20.

Figure 8a shows the removal rate curve of the straight heat pipe after polishing with PMMA and PS plastic abrasives. From the overall removal curve, the total removal efficiency of the PMMA abrasive is significantly higher than that of the PS abrasive, indicating that the PMMA abrasive can remove more satellites under the same conditions. At the same #200 particle size, the removal mass of the heat pipe treated with PMMA reached 0.043 g, while the removal mass of the PS abrasives was only 0.017 g, less than 40% of the removal mass of the PMMA abrasives. In stark contrast to these, it can be seen in Figure 4b that the removal mass of SiO_2_ particles reached about 0.2 g, which is not the same order of magnitude as the two kinds of plastic abrasives. The removal quality of the PMMA abrasives increases most significantly in the first 2 min of jet polishing, and the removal quality of the PMMA abrasives of three particle sizes in 0–2 min accounts for more than 60% of the total removal mass, indicating that most of the removal tasks have been completed in the early phase of jet polishing (within 2 min). This indicates that this processing method has a significant time advantage compared to other post-processing methods [37]. Overall, both plastic abrasives exhibit good cleaning ability. But for a better removal of these small defects, the PMMA-#200 abrasive is optimal.

**Figure 9:**
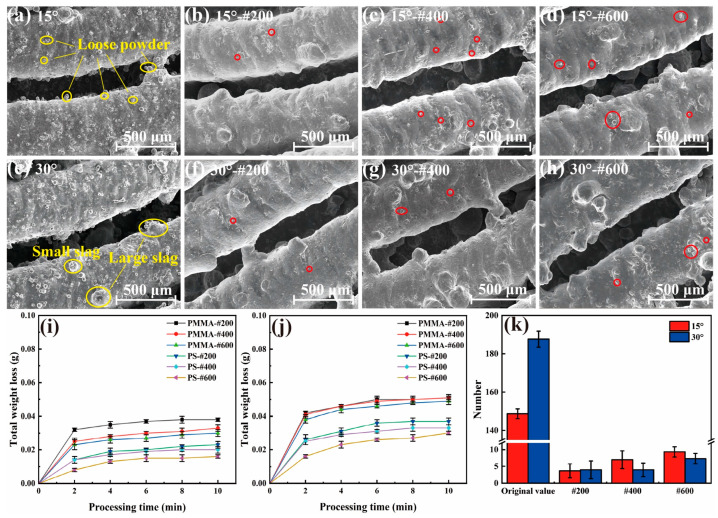
Morphology of the bent heat pipe (**a**,**e**) before and (**b**–**d**), (**f**–**h**) after AWJ; curves of weight loss of (**i**) 15° and (**j**) 30° heat pipes; and (**k**) the amount of residual structure in both angles.

In addition, we investigated the removal effect of the PMMA and PS abrasives on bent heat pipes, measuring the weight change, as shown in Figure 9i,j. Like the cleaning results of the straight heat pipes, the weight loss of the PMMA abrasives is all higher than the PS abrasives. When the bent angle of the heat pipe increases from 0° to 30° (0° means that the heat pipe is straight), the weight loss of both heat pipes increases, which is due to the increasing amount of printing defects during the printing of the structure in a larger tilt angle [22]. The cleaning results of the PMMA abrasive on the bent heat pipe are shown in Figure 9a–h. The particle count before and after treatment by different angles is shown in Figure 9k. The PMMA-#200 abrasive remains effective in treating the elbow. Only 1–3 scattered powders were found in a single counting area at the corner, and fewer than 10 satellites were observed on the inner surfaces of the #400 and #600 groups, significantly enhancing the cleanliness of the inner surface. As a result, the PMMA-#200 abrasive is considered the best choice for bent heat pipe cleaning, since no corner erosion was found either.

**Figure 10:**
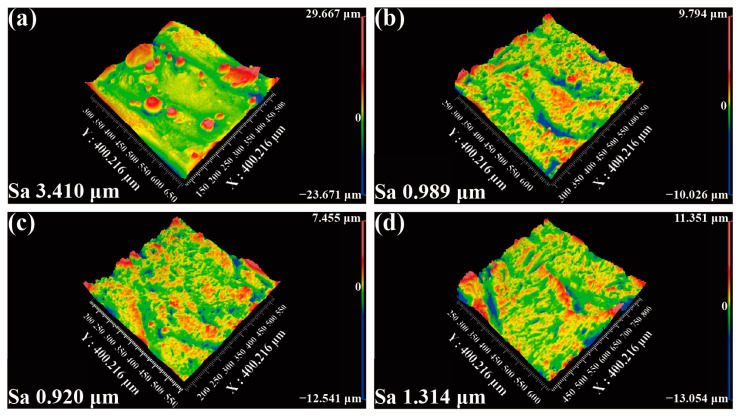
Second-half surface morphology of the heat pipe treated with the PMMA-#200 abrasive: (**a**) original surface, (**b**) 0°, (**c**) 15°, and (**d**) 30°.

A white light interferometer was used to observe the inner surface of the three angles of heat pipes treated with the PMMA-#200 abrasive. The surface roughness Sa of the straight heat pipes was improved from 3.410 μm to 0.989 μm. As shown in Figure 10, there are many small defects on the original surface. After the cleaning process is complete, the surface roughness is much lower. And the ripple marks left by the printing process are left behind, which is thought to facilitate heat transfer [38].

After the above analysis of the removal quality, time, residual floating powder, and morphology were observed under scanning electron microscopy and 3D optical surface profiler. In terms of abrasive types, the PMMA abrasive is more effective than the PS abrasive. Regarding particle size, the #200 particle size exhibits the best overall performance among the three particle sizes. Therefore, the use of #200 particle size PMMA abrasives for high-pressure water jet treatment can obtain the best treatment effect. This treatment removes the surface unstable structure at the same time, to ensure the integrity of the stable structure conducive to heat transfer, but it also has a very high processing efficiency, and can be completed in a few minutes for a 70 mm long straight heat pipe or bent heat pipe.

### 3.3. CFD Mechanism Explanation and Analysis

**Figure 11:**
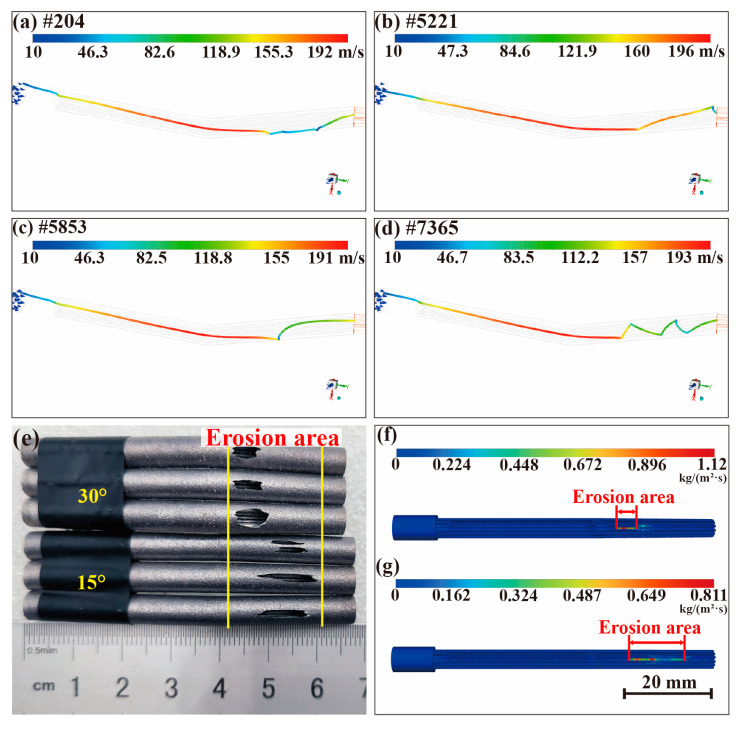
Erosion of heat pipe caused by SiO_2_ abrasives: (**a**–**d**) the motion path of the randomly selected abrasives, (**e**) erosion location of the heat pipe, and heat pipe simulation diagram of #600: (**f**) 30° and (**g**) 15°.

According to the results of the above experimental tests and characterization, the plastic abrasive effectively addresses the problem of corner erosion caused by the jet. In terms of removal rate, surface morphology, and residual floating powder, PMMA has a better treatment effect than PS, and PMMA-#200 has the best overall integrated effect. Next, in combination with the results of the simulation analysis, we further explored the mechanisms of jet removal and the factors that influence the erosion of SiO_2_ abrasive wear through simulation models. To explore the reasons why plastic abrasives do not erode and the PMMA abrasive has a better removal, CFD simulations were used to explain the core mechanism of abrasive water jet inside the bent heat pipe. In the case of a single particle, after the first collision of the particle carried by the jet in the flow field, the velocity of the particle will not be completely lost and stalled. Figure 11a–d show the velocity trajectories obtained from the simulations for four different abrasives. Firstly, after the collision, the kinetic of the different abrasives is consumed during the impact, transferring into the energy required for the material removal of the heat pipe. Secondly, the particles are accelerated in the flow field after collision. Subsequently, they continue to rise to a high or relatively high speed, and then impact the next unstable satellite or the inner wall of the heat pipe again, initiating a cycle of continuous acceleration, collision, and repetition of this process. Lastly, is the particles are ejected from the outlet of the heat pipe. In the jet process, numerous particles adhere to the accelerated collision law mentioned above. Different particles collide at distinct positions, times, and angles, leading to diverse erosion morphologies along the heat pipes at various locations.

**Figure 12:**
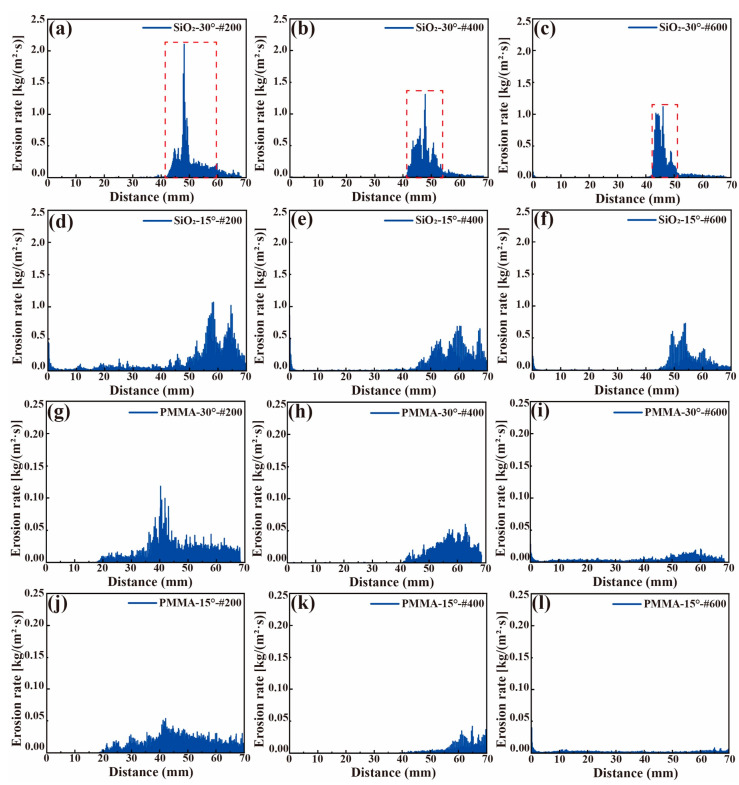
Erosion energy of the heat pipe caused by plastic abrasives: different sizes for (**a**–**f**) SiO_2_ and (**g**–**l**) PMMA abrasives.

For the SiO_2_ abrasives, after the accumulation of a large quantity of abrasive impingement, there is also the problem of corner erosion. Figure 11e–g show the erosion location of the heat pipe obtained experimentally and the erosive cloud image obtained using simulation. As can be seen, the heat pipes at different angles have different erosive morphologies. In Figure 11e, the erosion position of the 30° heat pipe is within 4–13 mm from the corner, while the erosion position of the 15° heat pipe is delayed to 12–23 mm, and the axial size of the erosion morphology is also changed from 5–6 mm to 8–15 mm. The erosion position obtained using simulation in Figure 11f,g is consistent with the actual position, and the difference in corner erosion for the 15° and 30° heat pipes can also be well explained by Formula (1).

(1) $$R_{erosion}=\sum_{p=1}^{N_{particle}}\frac{m_{p}C(d_{p})f(\alpha )v^{b(v)}}{A_{face}}$$

where *m_p_* is the mass of a particle, *C(d_p_)* is the particle size function, α is the impact angle between particle path and wall surface, *f(α)* is the impact angle function, v is the relative velocity of particles, *b(v)* is the particle relative velocity function, and *A_face_* is the area of the wall [39]. Under the same conditions, in the 30° bend, the relative velocity and impact angle of the abrasives is greater than 15°, so the erosion rate of 30° is greater than the 15° bend. Correspondingly, Figure 12a–c show that the 30° erosion curve has a higher peak value, indicating that the erosion energy is stronger. The size of the peak value of the energy and the law of concentrated distribution jointly cause the phenomenon that the 30° bent heat pipe is sprayed faster.

The position of this erosion morphology corresponds to the fact that the peak erosion area (The red box circled in Figure 12) of the 30° bend treated using the SiO_2_ abrasive is more concentrated and closer to the corner, whereas the peak erosion area of the 15° bend is longer and farther away from the corner. As the abrasive grain size increases, the peak value of the erosion gradually increases, but the location of the erosion does not change significantly. Figure 12 shows the axial distribution of the erosion rate of the heat pipe treated with the PMMA and SiO_2_ abrasives, respectively. There is a 17-fold difference in the magnitude of erosion between SiO_2_ and PMMA. There is also a very pronounced peak in the erosion curve of the SiO_2_ abrasive group, while the erosion of PMMA is spread over the entire length. The very low erosion energy and more uniform distribution over the length of the heat pipe make the PMMA abrasives less severe than the SiO_2_ abrasives, which is in high agreement with the actual macroscopic morphology of the heat pipe treated by both.

**Figure 13:**
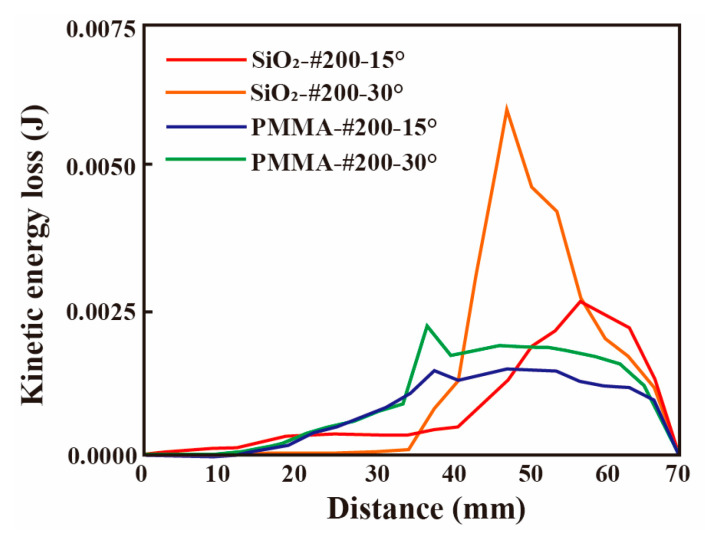
The kinetic energy loss diagram of the heat pipe treated with the PMMA and SiO_2_ abrasives in the jet direction.

As can be seen from Figure 13, the peak value of momentum loss in the y direction (the length direction of the heat pipe) of the 30° bent heat pipe treated with the SiO_2_ abrasives is more than twice that of 15°. This peak value at a specific position in the y direction also reveals the law of the erosion morphology, which proves that there is a sharp energy conversion at different positions of the 15° and 30° corners. The momentum of the particle deteriorates into the energy of the unstable floating powder structure removed, which results in the erosion effect of the PMMA abrasives on the heat pipe being much weaker than the SiO_2_ abrasives.

In addition, the physical properties of the abrasive particles, such as shape, size, and hardness, have an impact on erosion [30]. According to the data in Table 3, the density and hardness of PMMA are 1/2 and 1/3 those of SiO_2_. Because the SiO_2_ abrasive particles are irregular and have sharp edges and corners, while the PMMA abrasive particles are relatively regular and circular, the damage caused by the PMMA abrasive particles to the inner wall of the heat pipe will be smaller. Finally, it was determined that the PMMA abrasive particles can completely avoid erosion phenomena and can well remove satellites and floating powders.

Although both the PMMA and the PS abrasive particles are plastic abrasive particles, the removal of the PMMA abrasive particles with the same particle size is significantly better than that of the PS abrasive particles. This phenomenon is analyzed in combination with Formula (2).

(2) $$F_{D}=\frac{1}{2}\rho v^{2}C_{D}A$$

*F_D_* is the drag force, ρ is the mass density of the fluid, *v* is the flow velocity relative to the object, A is the reference area, and *C_D_* is the drag coefficient [40]. Compared to PS abrasives, PMMA abrasives have higher density and hardness. According to Formula (2), PMMA particles with the same particle size in the flow field can obtain greater drag force and thus have more energy for particle acceleration and wall processing. At the same time, the plastic abrasive particles have a certain water absorption within the acceptable range. The PMMA abrasive water absorption is stronger, the kinetic energy carried by a single particle is larger, the particles will have a certain expansion after water absorption, and this increase in volume will also lead more jet energy impact to the target surface, making the treatment effect better. Therefore, the PMMA abrasive particles finally show a better treatment effect.

According to the results from Figure 6, Figure 9 and Figure 10, the PMMA and PS abrasive particles do not exhibit signs of erosion. Thus, analyzing the experimental and CFD results, it is evident that the heat pipe treated with the PMMA-#200 abrasive particles ensures efficient removal without causing erosion to the inner wall. Therefore, selecting the PMMA-#200 abrasive particles is the optimal choice for addressing related issues.

In the future, our plans include initiating research on the treatment of heat pipes with more bending times and longer lengths. We also intend to explore the heat transfer performance of actual packaged heat pipes, the deformation of heat pipe grooves after a period of operation, and the presence of internal blockages that may affect heat transfer. Additionally, we will continue to investigate treatment methods for heat pipes made of materials other than AlSi10Mg and explore heat pipes with cross-section shapes other than those examined in this study.

## 4. Conclusions

To address the challenge of efficiently removing the unstable structure from the inner wall of an additive heat pipe while retaining the fluctuation plane to enhance heat transfer capacity, the feasibility and efficiency of using hard plastic abrasives to treat unstable structures with high-pressure water jets was studied. By combining experimental and simulation methods, three different abrasive particles of three sizes were used to treat the heat pipe at different angles using high-pressure jets. The impact of abrasive grain type and grain size on the inner surface of the treated heat pipe was investigated. The microscopic principles of abrasive particle velocity, trajectory, and energy were revealed by simulations. The reasons for the different macroscopic removal results were analyzed. Some conclusions could be made:

1. The SiO_2_ abrasive can effectively remove both satellites and balling defects, but will lead to corner erosion and the heat pipe leaking rapidly. For a 15° and 30° heat pipe, the leaking time is 15–30 s and 10–20 s, respectively, showing that the SiO_2_ abrasive is not suitable for cleaning bent heat pipes.
2. Simulation revealed that the reacceleration of the abrasive is key to the cleaning process. Further analysis found that the erosion caused by the SiO_2_ abrasive particles will be enhanced with the increase in the bent angle or in the abrasive particle size. At a 30° angle, the erosion rate is more concentrated at the corner, with a higher peak, resulting in a shorter leakage time.
3. The erosion rate of the SiO_2_ abrasive particles is 17 times higher than that of the PMMA abrasive particles, and the peak value of the momentum loss in the jet direction is more than 2 times higher. Second, the strength of PMMA in terms of physical properties such as density and hardness is much lower than that of the SiO_2_ abrasive particles, which, combined with its relatively homogeneous and rounded abrasive shape, makes it well suited for handling the microstructure of additively manufactured heat pipes.
4. The PMMA abrasive effectively eliminates satellites, and the removal efficiency of PMMA-#200 on the satellites is close to complete. The surface quality Sa of the straight heat pipe was increased from 3.410 μm to 0.989 μm after the PMMA-#200 abrasive treatment.

## Figures and Tables

**Table 1:** Printing parameters of heat pipes used in the experiment.

Process Parameters	Specific Content
Laser power	135 W
Spot size	70 μm
Scan speed	900 mm/s
Line spacing	0.12 mm
Layer thickness	0.03 mm

**Table 2:** Parameter settings for studying the effect of individual parameters.

Name	Specific Content
Jet pressure	30 MPa
Flow rate of water	15 L/min
Concentration of abrasive	1.5 wt.%
Size of abrasive	#200, #400, #600
Types of abrasive	SiO_2_, PMMA, PS

**Table 3:** Information on the physical properties of the three kinds of abrasive particles.

	SiO_2_	PMMA	PS
Density (g/cm^3^)	2.2	1.2	1.05
Mohs Hardness	7.0	2–2.5	2
Hygroscopicity	0	0.4%	0.02%

## Data Availability

Data are contained within the article.

## Appendix A

**Table A1:** Information on full-factorial experiments.

	Level 0	Level 1	Level 2
Factor A (Types of abrasive)	SiO_2_	PMMA	PS
Factor B (Size of abrasive)	#200	#400	#600
Factor C (Angle of heat pipe)	0°	15°	30°

**Table A2:** Design of full-factorial experiments.

		Factor C		
Factor A	Factor B	0	1	2
0	0	000	001	002
0	1	010	011	012
0	2	020	021	022
1	0	100	101	102
1	1	110	111	112
1	2	120	121	122
2	0	200	201	202
2	1	210	211	212
2	2	220	221	222
